# Integrated microRNA and mRNA Transcriptome Sequencing Reveals the Potential Roles of miRNAs in Stage I Endometrioid Endometrial Carcinoma

**DOI:** 10.1371/journal.pone.0110163

**Published:** 2014-10-17

**Authors:** Hanzhen Xiong, Qiulian Li, Shaoyan Liu, Fang Wang, Zhongtang Xiong, Juan Chen, Hui Chen, Yuexin Yang, Xuexian Tan, Qiuping Luo, Juan Peng, Guohong Xiao, Qingping Jiang

**Affiliations:** 1 Department of Pathology, The Third Affiliated Hospital, Guangzhou Medical University, Guangzhou, China; 2 Key Laboratory of Major Obstetrics Diseases of Guangdong Province, The Third Affiliated Hospital, Guangzhou Medical University, Guangzhou, China; University of Alabama at Birmingham, United States of America

## Abstract

Endometrioid endometrial carcinoma (EEC) is the most dominant subtype of endometrial cancer. Aberrant transcriptional regulation has been implicated in EEC. Herein, we characterized mRNA and miRNA transcriptomes by RNA sequencing in EEC to investigate potential molecular mechanisms underlying the pathogenesis. Total mRNA and small RNA were simultaneously sequenced by next generation sequencing technology for 3 pairs of stage I EEC and adjacent non-tumorous tissues. On average, 52,716,765 pair-end 100 bp mRNA reads and 1,669,602 single-end 50 bp miRNA reads were generated. Further analysis indicated that 7 miRNAs and 320 corresponding target genes were differentially expressed in the three stage I EEC patients. Six of all the seven differentially expressed miRNAs were targeting on eleven differentially expressed genes in the cell cycle pathway. Real-time quantitative PCR in sequencing samples and other independent 21 pairs of samples validated the miRNA-mRNA differential co-expression, which were involved in cell cycle pathway, in the stage I EEC. Thus, we confirmed the involvement of hsa-let-7c-5p and hsa-miR-99a-3p in EEC and firstly found dysregulation of hsa-miR-196a-5p, hsa-miR-328-3p, hsa-miR-337-3p, and hsa-miR-181c-3p in EEC. Moreover, synergistic regulations among these miRNAs were detected. Transcript sequence variants such as single nucleotide variant (SNV) and short insertions and deletions (Indels) were also characterized. Our results provide insights on dysregulated miRNA-mRNA co-expression and valuable resources on transcript variation in stage I EEC, which implies the new molecular mechanisms that underlying pathogenesis of stage I EEC and supplies opportunity for further in depth investigations.

## Introduction

Endometrial cancers are the most frequent cancer occurring in the female genital tract, and its incidence has increased in recent years [1]. Endometrioid endometrial carcinoma (EEC) is the most dominant subtype, accounting for ∼80% of cases [2]. To date, most clinical trials of chemotherapeutics for advanced and recurrent EEC have shown limited benefits [3], [4] and information regarding the molecular mechanisms of EEC etiology is still limited. Searching for innovative diagnosis markers and therapeutic targets for EEC is thus necessary.

Aberrant transcript expression levels are commonly observed in cancer and these aberrations could alter biological pathways and disease phenotypes. Next-generation sequencing (NGS) of RNA (RNA-seq) is a powerful tool to investigate the comprehensive transcriptome. RNA-seq has greatly enhanced our knowledge of the transcriptome in cancer [5] recently. MicroRNAs (miRNAs) are a class of small non-coding RNAs that can regulate mRNA expression and control various biological processes [6] through binding to the 3′ untranslated region of mRNAs. It is reported that about 30% of human genes [7] and virtually all cellular processes are regulated by miRNAs [8]. Studies on miRNA dysregulation in cancers have risen rapidly recently, including those in EEC. MiRNAs such as hsa-miR-503, hsa-miR-205, and hsa-miR-200b are dysregulated in EEC and they could regulate cell proliferation, differentiation, apoptosis, and carcinogenesis [9]–[11]. However, studies on concurrent transcriptome characterization of both mRNA and miRNA in EEC are still lacking.

In this study, we applied an integrative approach by simultaneously sequencing both miRNA and mRNA for International Federation of Gynecology and Obstetrics (FIGO) Stage I EEC and adjacent non-tumorous tissues to investigate the mechanisms responsible for the pathogenesis of EEC. Quantitative real-time reverse transcription PCR (qRT-PCR) in other independent patients was performed on the selected miRNA and mRNAs. Transcript sequence variants such as single nucleotide variant (SNV) and short insertions and deletions (Indels) were also characterized. Our investigation may shed light on the molecular pathogenesis of EEC and offer new possibilities for early diagnosis and systemic treatment.

## Materials and Methods

### Ethics Statement

Our study design received approval from the institutional review board of the third affiliated hospital of Guangzhou medical college (Guangzhou, China). Written informed consent was obtained from all patients.

### Sample collection and RNA extraction

Three female patients aged from 32 to 47 were diagnosed as FIGO stage I EEC (two stage IA patients and one stage IB patient) in the third affiliated hospital of Guangzhou medical college. All primary tumor and adjacent non-tumorous samples were obtained from these three patients who underwent surgical tumor resection. All samples were frozen at −80°C until RNA extraction. Total RNA was isolated by using RecoverAll Total Nucleic Acid Isolation Kit (Life Technologies, Carlsbad, CA, USA) for mRNA and miRNA sequencing, according to the manufacturer’s instructions. Integrity of RNA was checked by Agilent 2100 bioanalyzer.

### Sequencing

The sequencing library was prepared according to the standard protocol. Briefly, for mRNA sequencing, total RNA was firstly poly-A-selected followed by fragmentation of RNA into small pieces. The cleaved RNA fragments were reverse transcribed to cDNA end-repaired and ligated with Illumina adapters using Quick ligation TM kit (NEB) and DNA ligase. The libraries were then fractionated on agarose gel; 200-bp fragments were excised and amplified by PCR. After purification, the quality of libraries was checked by using Bioanalyzer 2100 (Agilent). MRNA sequencing was then performed on an Illumina HiSeq 2000 sequencer with 100 bp pair-end reads. For small RNA sequencing, libraries were prepared by ligating different adaptors to the total RNA followed by reverse transcription and PCR amplification. Small RNA libraries were sequenced on the Illumina HiSeq 2000 sequencer with 50 bp single-end reads, according to the standard manufacturer’s protocol. All raw data have been deposited in the NIH Short Read Archive database (SRP045645).

### Reads processing

Raw mRNA sequencing reads were filtered for adapters and ribosomal RNA. Reads containing five or more positions with a quality score less than 19 were also removed from further analysis. Remained high-quality sequencing reads were then aligned to human genome (hg19) by using Tophat [12]. The matched reads were aligned to Human transcriptome (Ensembl, GRCh37.73). Cufflinks [13] was used to calculate mRNA expression level which was measured by RPKM (reads per kilobase per million).

For small RNA sequencing reads, all low quality reads, such as reads with adapter contaminants or poly A sequences were filtered. Sequences shorter than 18 nt after trimming adapters were removed. The high-quality clean reads were mapped to o the human genome using the bowtie software [14]. Small RNA tags were aligned to the miRNA precursor/mature miRNA in miRBase [15] (release 20). To identify small RNA tags originating from rRNA, tRNA, snRNA, and snoRNA, NCBI GenBank data and Rfam data were used.

We applied the pair wise t-test to filter differentially expressed miRNAs and mRNAs for the two groups. False discovery rate (FDR)-adjusted P values (P<0.05) and absolute fold change >1 were set as the cutoff.

### MiRNA target prediction

The miRNA target prediction was done by three current available methods: Targetscan [16], PITA [17], and miRanda [18]. For each prediction method, high efficacy targets were selected by the following criteria: (1) Targetscan: sum of context score −0.2; (2) PITA: ΔΔG≤−10 kcal/mol; (3) miRanda: score ≥155 and energy ≤−20 kcal/mol. Predicted miRNA–mRNA pairs were further selected according to the integrating mRNA and miRNA transcript expression data.

### QRT-PCR of miRNA and mRNA expression

Expression of selected miRNAs and mRNAs were validated by qRT-PCR. Another 21 pairs of samples from stage I EEC patients (12 stage IA and 9 stage IB patients) were collected in addition to the original three pairs of sequencing samples. For mRNA expression, 2 µg total RNA was reverse transcribed and QPCR was done in the real-time cyclers (Strata Gene MX3000P qPCR system). The relative fold changes were determined using the 2^−ΔΔCt^ method with GAPDH as endogenous controls.

For miRNA expression, 5 ng of total RNA per miRNA was reverse transcribed into cDNA with SYBR-Green PCR Master Mix (Takara). QPCR was done in real-time cyclers (Strata Gene MX3000P qPCR system). The relative expression levels were determined using the 2^−ΔΔCt^ method with U6 small nuclear RNA as endogenous controls.

Each reaction was done in triplicate. Pair wise t-test was used to determine the expression difference between the two groups.

### SNV/Indels detection

SNV/Indels detection was carried out by using VarScan [19] based on the following filtering criteria: 1) depth of coverage over 3×; 2) variation frequency over 20%; 3) base quality over 20. Detected SNVs and Indels were then annotated by using ANNOVAR [20].

## Results

### Overview of transcriptome sequencing results

To reveal the transcriptome of EEC, we sequenced the mRNA and small RNA from the same total RNA samples of stage I EEC patients using Illumina HiSeq 2000 sequencer. From the six mRNA libraries, an average of 52,716,765 pair-end 100 bp clean reads was generated (Table 1). Six small RNA libraries were also sequenced and 11,669,602 single-end 50 bp clean reads were generated on average (Table 2).

**Table 1:** Summary of mRNA sequencing.

Sample id	Clean reads	Mapped reads	Uniquely mapped reads	Paired mapped reads	Unmapped reads	Transcript coverage (×)
S1_N	53,204,178	47,891,621	45,603,601	44,892,334	5,312,557	40.63
S1_T	54,527,132	49,915,956	47,689,962	47,086,014	4,611,176	42.46
S2_N	52,006,058	47,807,313	46,016,910	45,189,796	4,198,745	40.82
S2_T	52,254,225	48,152,928	45,232,887	45,521,896	4,101,297	40.51
S3_N	52,891,237	48,016,297	45,448,760	45,323,990	4,874,940	40.61
S3_T	51,417,759	46,261,459	43,257,947	43,832,254	5,156,300	38.94
Average	52,716,765	48,007,596	45,541,678	45,307,714	4,709,169	40.66

**Table 2:** Summary of miRNA sequencing.

ID	Clean reads	miRNA	rRNA	snoRNA	tRNA	Mapped reads	Unmapped reads
S1_N	12,426,115	12,385,299	36,994	1,553	2,269	10,531,371	1,894,744
S1_T	11,041,505	10,994,036	42,878	1,549	3,042	9,433,794	1,607,711
S2_N	10,571,133	10,557,967	11,476	136	1,554	8,905,234	1,665,899
S2_T	10,849,264	10,815,517	31,412	969	1,366	7,978,261	2,871,003
S3_N	14,584,595	14,522,078	60,098	635	1,784	9,908,455	4,676,140
S3_T	10,544,999	10,424,664	111,207	2,038	7,090	7,158,492	3,386,507
Average	11,669,602	11,616,594	49,011	1,147	2,851	8,985,935	2,683,667

**Figure 1:**
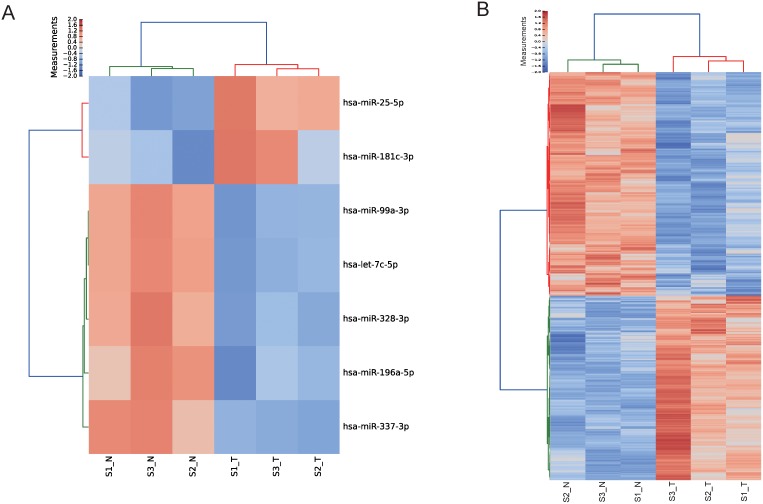
Hierarchical clustering of differentially expressed miRNAs and mRNAs in endometrioid endometrial carcinoma (S1_T, S2_T, S3_T) and adjacent non-tumorous tissues (S1_N, S2_N, S3_N).

A total of 1079 genes were differentially expressed, including 488 up regulated and 591 down regulated ones (Figure 1). Seven miRNAs were differentially expressed. Among them, hsa-miR-99a-3p, hsa-let-7c-5p, hsa-miR-196a-5p, hsa-miR-328-3p, hsa-miR-337-3p were down regulated while hsa-miR-181c-3p and hsa-miR-25-5p were up regulated (Figure 1).

### MiRNA target prediction and integration of mRNA and miRNA expression profiles

We ran the three prediction algorithms: Targetscan [16], PITA [17], and miRanda [18] using the selected 7 miRNAs and 1079 genes as input. This resulted in 1098 miRNA-mRNA pairs, which were supported by at least two prediction algorithms. We further integrated the sequencing data into the predicted miRNA-mRNA pairs to validate the target pairs. A total of 438 target pairs were found inversely expressed, including 320 dysregulated genes.

### Functional enrichment

Biological pathway and gene ontology (GO) enrichment were done for the 320 dysregulated genes. Pathway analysis identified 12 pathways overrepresented with dysregulated miRNA target genes. Overall, a genetic cluster summarizing the functions of Cell cycle (hsa04110) was found to have the highest relationship with EEC (Table 3). Considering the pathways involved in cancer development, we identified several significantly related pathways, including p53 signaling pathway, GnRH signaling pathway, Pathways in cancer and Gap junction (Table 3). In addition to these classical pathways, pancreatic cancer and thyroid cancer pathway were also overrepresented with dysregulated genes, implicating a common oncogenic basis. GO enrichment analyses indicated that 9 GO items were significantly enriched with the dysregulated genes (Table 4). Consistent with the pathway analysis, the most significant GO category is mitotic cell cycle (Table 4).

**Table 3:** Pathway analysis based on miRNA-targeted differentially expressed genes.

KEGG id	KEGG description	KEGG subclass	*p*-value
hsa04110	Cell cycle	Cell growth and death	1.21E-03
hsa04115	p53 signaling pathway	Cell growth and death	1.44E-02
hsa04912	GnRH signaling pathway	Endocrine system	2.21E-02
hsa04713	Circadian entrainment	Environmental adaptation	2.24E-02
hsa05200	Pathways in cancer	Cancers	3.41E-02
hsa04973	Carbohydrate digestion and absorption	Digestive system	3.94E-02
hsa0480	Glutathione metabolism	Metabolism of other amino acids	4.03E-02
hsa05212	Pancreatic cancer	Cancers	4.09E-02
hsa04540	Gap junction	Cell communication	4.32E-02
hsa04114	Oocyte meiosis	Cell growth and death	4.65E-02
hsa05216	Thyroid cancer	Cancers	4.72E-02
hsa04918	Thyroid hormone synthesis	Endocrine system	4.90E-02

**Table 4:** Gene ontology analysis based on miRNA-targeted differentially expressed genes.

GO id	GO description	GO class	*p*-value
GO:0000278	mitotic cell cycle	Process	1.60E-03
GO:0000777	condensed chromosome kinetochore	Component	1.17E-02
GO:0007051	spindle organization	Component	1.17E-02
GO:0008156	negative regulation of DNA replication	Process	1.17E-02
GO:0032133	chromosome passenger complex	Component	2.17E-02
GO:0005876	spindle microtubule	Process	2.17E-02
GO:0009636	response to toxic substance	Process	2.17E-02
GO:0051301	cell division	Component	4.72E-02
GO:0007067	mitosis	Component	4.72E-02

### QRT-PCR validation of miRNA and mRNA co-expression

**Figure 2:**
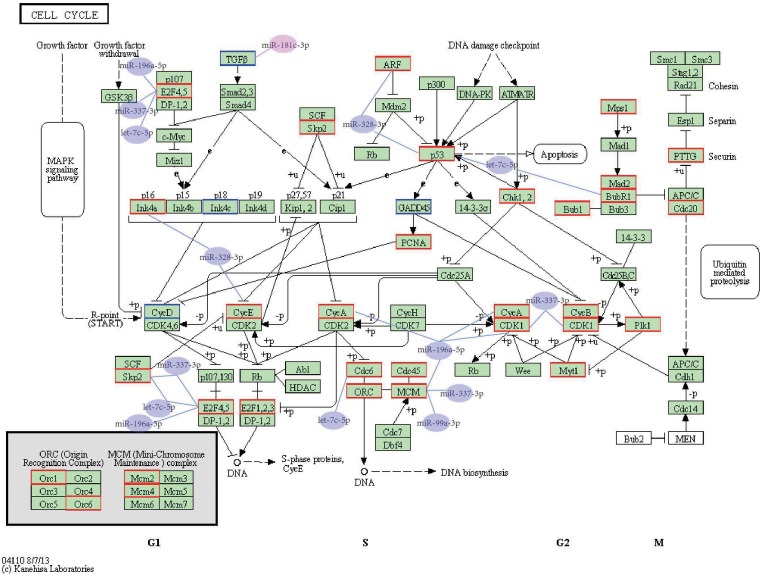
MiRNA-gene network of the cell cycle pathway in endometrioid endometrial carcinoma. Protein symbols were marked according to gene expression pattern. Those with red frames are up regulated while those with blue frames are down regulated in endometrioid endometrial carcinoma tissues. Similarly, miRNAs with blue background were down regulated while the miRNA with red ground were up regulated in endometrioid endometrial carcinoma tissues.

**Figure 3:**
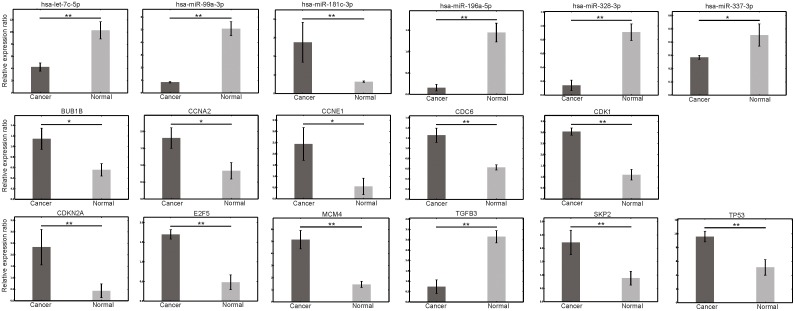
QPCR expression on QPCR-validated genes and miRNAs in the cell cycle pathway. *: *p*<0.05; **: *p*<0.01.

Our pathway analysis showed that the most significant pathway overrepresented with dysregulated miRNA target genes was the cell cycle pathway (*P* = 1.21E-03). Since human carcinogenesis is believed to result from uncontrolled cell proliferation and many molecules in the cell cycle pathway have been reported to be associated with endometrial cancer [21], [22], our findings prompted us to validate the expression of miRNAs and their potential target genes in the cell cycle pathway. As shown in Figure 2, among the significantly dysregulated miRNAs, 5 down regulated miRNAs (hsa-let-7c-5p, hsa-miR-196a-5p, hsa-miR-328-3p, hsa-miR-337-3p, and hsa-miR-99a-3p) were predicted to target genes encoding 10 upregulated cell cycle pathway-related mRNAs (*E2F5, CDKN2A, CCNA2, TP53, BUB1B, CCNE1, CDK1, MCM4, SKP2,* and *CDC6*). Additionally, one upregulated miRNA, hsa-miR-181c-3p, was predicted to target the downregulated *TGFB3* gene in cell cycle pathway (Figure 2). All these 6 miRNAs and 11 genes were present in the three pairs of sequencing samples. Differential expression was confirmed for all the 6 miRNAs and 11 genes in sequencing samples and other independent 21 pairs of samples, as shown in Figure 3. These 6 miRNAs may therefore play a role in the pathogenesis of EEC.

In addition, for these 6 miRNAs and 11 mRNAs, synergistic regulations among the six miRNAs were detected (Figure 2). For example, *E2F5* was predicted as a target of hsa-miR-196a-5p, hsa-miR-337-3p and hsa-let-7c-5p; *TP53* was predicted as the target of both hsa-miR-328-3p and hsa-let-7c-5p; *MCM4* was targeted by hsa-miR-196a-5p, hsa-miR-337-3p, and hsa-miR-99a-3p; *SKP2* was targeted by hsa-miR-337-3p and hsa-let-7c-5p; *CDK1* was targeted by hsa-miR-196a-5p and hsa-miR-337-3p.

### Sequence variants in EEC transcriptome

Sequence variants analysis detecting SNVs and Indels was carried out using VarScan [19]. The results are summarized in Table 5. A total of 346 variants (17.06%) are from exonic regions of the genome while 1682 (82.93%) are from the non-protein coding regions. Around 75% (260/346) of the exonic variants, including 181 nonsynonymous SNVs, 14 stop gain variants and 65 frameshift Indels, would result in protein coding alterations. Moreover, 17 out of 260 protein-affected variants overlapped with the somatic mutations in the cancer (COSMIC) [23] (Table S1 and Table S2). Of the non-protein coding variants, around half (863/1682) are in the UTR region.

**Table 5:** Sequence variants identified from transcriptome data.

Variants	S1		S2		S3		Total	
	SNV	Indel	SNV	Indel	SNV	Indel	SNV	Indel
exonic	95	27	81	26	89	28	264	82
synonymous SNV	26	0	26	0	22	0	74	0
nonsynonymous SNV	67	0	50	0	65	0	181	0
stopgain	2	3	5	1	2	1	9	5
frameshift insertion	0	15	0	13	0	10	0	38
nonframeshift insertion	0	1	0	2	0	1	0	5
frameshift deletion	0	8	0	7	0	12	0	27
nonframeshift deletion	0	0	0	3	0	4	0	7
splicing	1	0	0	1	0	1	1	2
UTR3	209	59	201	70	180	80	587	209
UTR5	23	10	8	9	16	1	47	20
downstream	28	4	30	3	16	2	74	9
upstream	5	0	2	1	5	0	12	1
intronic	185	19	189	27	38	4	404	50
intergenic	114	11	92	8	41	5	244	25
Total	659	130	603	145	383	121	1630	398

## Discussion

Compared with traditional microarrays, NGS enables the identification of novel and more detailed studies of biological pathways in complex diseases. Here with mRNAs and small RNA sequencing for three pairs of stage I EEC and adjacent normal tissues, we provide a global overview of the stage I EEC transcriptome and reveal the miRNA regulation of transcript expression.

MiRNA data analyses identified 7 dysregulated miRNAs (Figure 1) and mRNA sequencing identified a total of 1079 differentially expressed genes (Figure 1). To gain insights into the targets of differentially expressed miRNAs, a stringent prediction of miRNA targets was made by using three different target prediction methods. We further integrated the sequencing data into the predicted miRNA-mRNA pairs to validate the target pairs. A total of 438 target pairs were found inversely expressed, which can serve as references for further studies on miRNA and its targets. Both GO and KEGG pathway enrichment analyses of the putative target genes revealed the dysregulation of the cell cycle process, suggesting that our results were in line with the basic tumor characteristics. We further validated the expression levels of miRNA-target pairs in the cell cycle pathway. As shown in Figure 2, 5 down regulated miRNAs (hsa-let-7c-5p, hsa-miR-196a-5p, hsa-miR-328-3p, hsa-miR-337-3p, and hsa-miR-99a-3p) were predicted to target genes encoding 10 upregulated cell cycle pathway-related mRNAs (*E2F5, CDKN2A, CCNA2, TP53, BUB1B, CCNE1, CDK1, MCM4, SKP2,* and *CDC6*). Additionally, one upregulated miRNA, hsa-miR-181c-3p, was predicted to target the downregulated *TGFB3* gene. All these 6 miRNAs have previously been suggested to be involved in human cancers. Downregulation of hsa-let-7c has been identified in multiple cancer types, such as non-small cell lung cancer [24], pancreatic cancer [25], prostate cancer [26]. Moreover, let-7c was also found to be down-regulated in endometrial sarcomas when compared to the control samples [27]. Expression of miR-196a was also found to be significantly down-regulated in malignant melanoma cell lines and tissue samples [28]. Decreased expression of miR-328 was also found in multiple cancer types [29], [30]. Loss of has-miR-337-3p expression has been associated with lymph node metastasis of human gastric cancer [31]. Downregulation of miR-99a was discovered in both plasma and tissues of endometrial cancer patients [32]. Overexpression of miR-181c has also been reported in other cancers, such as gastric cancer [33]. In the current study, we confirmed the involvement of hsa-let-7c-5p and hsa-miR-99a-3p in EEC and firstly found dysregulation of hsa-miR-196a-5p, hsa-miR-328-3p, hsa-miR-337-3p, and hsa-miR-181c-3p in EEC. Since all these miRNAs are dysregulated in stage I EEC, they may serve as potential diagnostic markers in clinical practice. In addition, considering the inhibition effects on cancer growth or metastasis of hsa-let-7c [24], [26], hsa-miR-328 [29], hsa-miR-337 [31], these three miRNAs may provide new avenues for the prognosis and therapy for EEC. Among the target genes of these miRNAs in the cell cycle pathway, *TP53* [34], *CDKN2A* [34], *SKP2* [35], [36], *CCNE1* [36], *MCM4* [36], CDK1 [37] and *TGFB3* [38] have been extensively studied as anticancer targets in endometrial cancer. Overexpression of *CCNA2* was reported to be an indicator of a poor prognosis endometrial carcinoma [39]. Currently, there is no report about the relationship between *E2F5*, *BUB1B*, or *CDC6* and EEC. E2F5 is a transcription factor predicted as a target of hsa-miR-196a-5p, hsa-miR-337-3p and hsa-let-7c-5p. Human *E2F5* gene is oncogenic [40] and overexpression of this gene was associated with worse clinical outcome in other cancers [41]. Both *BUB1B* and *CDC6* are predicted as a target of hsa-let-7c-5p. *BUB1B* encodes a kinase involved in spindle checkpoint function and involvements of these two genes have been reported in other cancers [42], [43]. Their biological functions in EEC need further study. Moreover, based on our validated co-expression relationships of these miRNA-mRNA pairs, further *in* *vitro* experiments may be carried out to further confirm that the differentially expressed miRNAs synergistically affect the expression of the predicted targets.

In addition, for the miRNAs and mRNAs we validated in the cell cycle pathway, synergistic regulations among the six miRNAs were detected (Figure 2). MiRNA–miRNA synergism is important to understand the mechanism of complex post-transcriptional regulations in human [44]. Our results here may provide guide for further validation studies of the regulation mechanisms of these miRNAs.

In addition to expression analysis, we also analyzed the sequence variants. Besides proteins encoding SNV/Indels, a large number of variants are located in UTRs whose function still remains unknown (Table 5). The analysis of SNV, Indels may provide possible targets for further mechanistic studies.

In summary, here we report a comprehensive characterization of mRNA and miRNA transcriptome in stage I EEC from expression level, miRNA/mRNA regulations and sequence variation. We integratively analyzed differentially expressed miRNAs and their target genes. Differential expression levels of target genes in the cell cycle pathway and their regulatory miRNAs were further validated in other independent samples. Our results will provide a valuable reference for future studies in transcript variation and regulation and will potentially be useful for mechanistic and preclinical studies.

## Supporting Information

Table S1 **Detail information of the identified single nucleotide variants.**
(XLSX)

Table S2 **Detail information of the identified short insertions and deletions.**
(XLSX)
